# Assessing the accuracy of machine-assisted abstract screening with DistillerAI: a user study

**DOI:** 10.1186/s13643-019-1221-3

**Published:** 2019-11-15

**Authors:** Gerald Gartlehner, Gernot Wagner, Linda Lux, Lisa Affengruber, Andreea Dobrescu, Angela Kaminski-Hartenthaler, Meera Viswanathan

**Affiliations:** 1 RTI International–University of North Carolina Evidence-based Practice Center, Research Triangle Park, NC USA; 2 Department for Evidence-based Medicine and Evaluation, Danube University Krems, Krems, Austria; 3 Department of Family Medicine, Care and Public Health Research Institute (CAPHRI), Maastricht University, Maastricht, The Netherlands

**Keywords:** Systematic reviews, Machine-learning, Rapid reviews, Accuracy, Methods study

## Abstract

**Background:**

Web applications that employ natural language processing technologies to support systematic reviewers during abstract screening have become more common. The goal of our project was to conduct a case study to explore a screening approach that temporarily replaces a human screener with a semi-automated screening tool.

**Methods:**

We evaluated the accuracy of the approach using DistillerAI as a semi-automated screening tool. A published comparative effectiveness review served as the reference standard. Five teams of professional systematic reviewers screened the same 2472 abstracts in parallel. Each team trained DistillerAI with 300 randomly selected abstracts that the team screened dually. For all remaining abstracts, DistillerAI replaced one human screener and provided predictions about the relevance of records. A single reviewer also screened all remaining abstracts. A second human screener resolved conflicts between the single reviewer and DistillerAI. We compared the decisions of the machine-assisted approach, single-reviewer screening, and screening with DistillerAI alone against the reference standard.

**Results:**

The combined sensitivity of the machine-assisted screening approach across the five screening teams was 78% (95% confidence interval [CI], 66 to 90%), and the combined specificity was 95% (95% CI, 92 to 97%). By comparison, the sensitivity of single-reviewer screening was similar (78%; 95% CI, 66 to 89%); however, the sensitivity of DistillerAI alone was substantially worse (14%; 95% CI, 0 to 31%) than that of the machine-assisted screening approach. Specificities for single-reviewer screening and DistillerAI were 94% (95% CI, 91 to 97%) and 98% (95% CI, 97 to 100%), respectively. Machine-assisted screening and single-reviewer screening had similar areas under the curve (0.87 and 0.86, respectively); by contrast, the area under the curve for DistillerAI alone was just slightly better than chance (0.56). The interrater agreement between human screeners and DistillerAI with a prevalence-adjusted kappa was 0.85 (95% CI, 0.84 to 0.86%).

**Conclusions:**

The accuracy of DistillerAI is not yet adequate to replace a human screener temporarily during abstract screening for systematic reviews. Rapid reviews, which do not require detecting the totality of the relevant evidence, may find semi-automation tools to have greater utility than traditional systematic reviews.

## Background

A crucial step in any systematic review is the selection of relevant abstracts. To reduce the risk of falsely excluding relevant studies, methodological guidance recommends a dual-screening process [1, 2]. Two reviewers independently determine the eligibility of each record based on a predetermined list of inclusion and exclusion criteria. In its landmark document *Finding What Works in Healthcare: Standards in Systematic Reviews*, the US Institute of Medicine explicitly favors high sensitivity of literature searches and literature screening over high specificity [3].

Screening titles and abstracts, however, is a lengthy and labor-intensive process. Systematic reviewers often need to screen thousands of irrelevant abstracts to identify a few relevant studies. A cost-effectiveness analysis estimated that screening 5000 references takes 83 to 125 h per reviewer at a cost of approximately £13,000 (2013 prices; about 17,000 $US) [4].

In recent years, web applications that employ natural language processing technologies to support systematic reviewers during abstract screening have become more user-friendly and more common. In 2015, a systematic review by O’Mara-Eves and colleagues identified 44 studies addressing the use of text mining to reduce the screening workload in systematic reviews [5]. Commonly used tools that systematic reviewers can use without additional programming include Abstrackr [6], DistillerAI [7], EPPI (Evidence for Policy and Practice Information) Reviewer [8], RobotAnalyst [9], Rayyan [10], and/or SWIFT (SciOme Workbench for Interactive computer-Facilitated Text-mining)-Review [11]. These text-mining approaches use pattern recognition algorithms to predict the probabilities of record relevance or irrelevance. Text mining describes the process of filtering knowledge from unstructured data such as text. In the context of abstract screening, text mining is combined with text classification, which is the decision about the inclusion or exclusion of a given record [12, 13]. Applications that combine text mining with machine learning have the advantage of improving the system’s performance continuously. Consequently, the machine continuously adapts its decision rules based on human screeners’ decisions.

Such semi-automated screening tools can increase efficiency by reducing the number of abstracts needed to screen or by replacing one screener after adequately training the algorithm of the machine [14]. Savings in workload between 30 and 70% might be possible with the use of text-mining tools in systematic reviews [5]. The downside of the use of such tools, however, is that none of these tools has perfect sensitivity and a reduction in workload might be accompanied by missing relevant studies [5].

To date, several semi-automated screening tools have been validated [9, 11, 15–17]. Most research publications on this topic, however, have been produced by computer scientists and experts in medical informatics and artificial intelligence. Often studies have been conducted under highly controlled conditions using artificial bibliographic datasets. Furthermore, validation studies mostly used decisions about inclusion or exclusion at an abstract screening stage as a reference standard. Human decisions during abstract screening, however, vary and are an imperfect reference standard.

The goal of our project was to conduct a case study to explore a screening approach that temporarily replaces a human screener with a semi-automated screening tool. We were also interested in comparing the performance of this approach with that of single-reviewer screening and screening of abstracts by a semi-automated screening tool without human involvement after training the tool. Table 1 summarizes commonly used terms in this manuscript.

**Table 1:** Definitions of commonly used terms

Accuracy: the proportion of correctly classified records: $\frac{\left(\mathrm{TP}+\mathrm{TN}\right)}{\left(\mathrm{TP}+\mathrm{FP}+\mathrm{TN}+\mathrm{FN}\right)}$ False negatives (FNs): the number of records incorrectly classified as excludes. Also referred to as “missed studies.”	
False positives (FPs): the number of records incorrectly classified as includes.	
Prediction: a forecast of whether a record is relevant (include) or irrelevant (exclude) for a given systematic review.	
Semi-automated screening tool: any web-based application that employs a combination of text mining and text classification to assist systematic reviewers during the title and abstract screening process.	
Sensitivity: the ability of a screening tool to correctly classify *relevant* records as includes: $\frac{\mathrm{TP}}{\left(\mathrm{TP}+\mathrm{FN}\right)}$	
Specificity: the ability of a screening tool to correctly classify irrelevant records as excludes: $\frac{\mathrm{TN}}{\left(\mathrm{TN}+\mathrm{FP}\right)}$	
Text classification: a standard machine-learning process in which the aim is to categorize texts into groups of interest [18].	
Text mining: the process of discovering knowledge and structure from unstructured data.	
True negatives (TNs): the number of records correctly identified as excludes.	
True positives (TPs): the number of records correctly identified as includes.	

## Methods

The objective of our study was to assess the accuracy of an abstract screening approach that temporarily replaces one human screener with a semi-automated screening tool. To address our objective, we employed a diagnostic framework approach with a reference (gold) standard comparator group.

We chose DistillerAI as a semi-automated screening tool for our project. DistillerAI is a natural language processing tool within DistillerSR (www.evidencepartners.com/products/distillersr-systematic-review-software), a specialized, commercially available web-based software to conduct systematic reviews. DistillerAI offers a naïve Bayesian approach or a support vector machine classifier to screen abstracts after learning from manual screening. The naïve Bayesian approach provides probabilistic prediction scores regarding the inclusion or exclusion of records (0.5 is an inconclusive score). Prediction scores larger than 0.5 indicate a greater probability of a record being relevant rather than irrelevant; scores smaller than 0.5 indicate the opposite.

The support vector machine classifier offers nonprobabilistic, binary classifications (include, exclude, or cannot decide). It uses data from the training set to build a model that classifies new records as relevant or irrelevant. We chose DistillerAI as a screening tool for our project because it provides optimal flexibility regarding data import and export and an efficient technical helpline.

### Reference standard

We used data from an Agency of Healthcare Research and Quality (AHRQ) systematic review on pharmacological and nonpharmacological interventions for the treatment of depression as the reference standard [19]. For the purpose of this project, we focused on a single Key Question, which included 42 randomized controlled trials (RCTs). Because the scope was narrower than that of the original review, we replicated a targeted literature search with a focus on the Key Question of interest (comparative effectiveness). We searched PubMed and Embase because we knew from a bibliographic analysis that the 42 RCTs included in the report are indexed in these databases. We adapted the original search strategy of the AHRQ report and limited searches to the same period that the report had covered (1995 to 2015).

### Outline of general approach

**Fig. 1:**
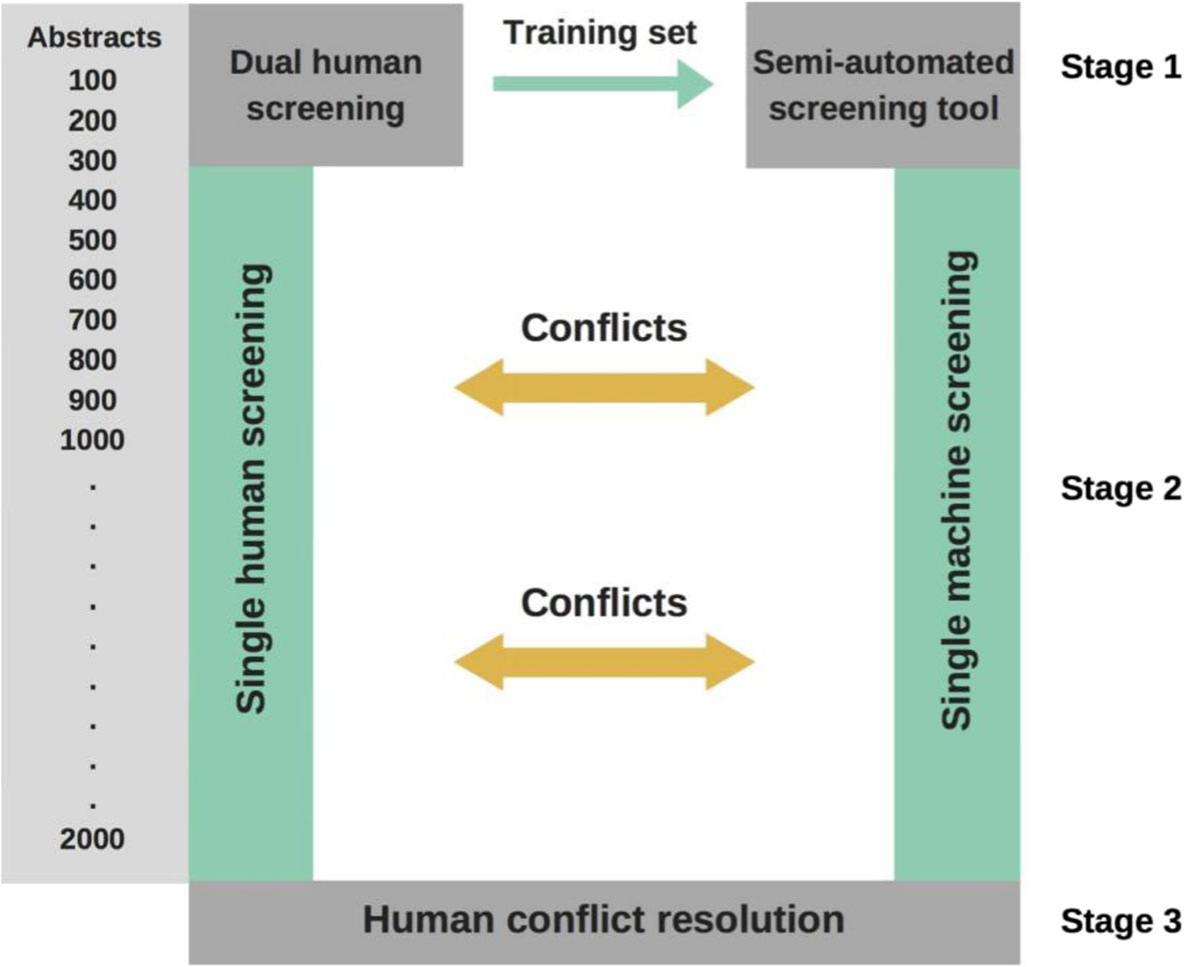
Graphical presentation of the study flow

Figure 1 depicts the screening approach in which the semi-automated screening tool temporarily replaced one human screener. Five independent teams applied this approach in parallel on the same topic. Teams consisted of professional systematic reviewers with extensive experience in literature screening and evidence syntheses.

Stage 1 mimicked a regular dual-reviewer abstract screening process. After a pilot phase with 50 records to calibrate screeners, 2 reviewers independently screened abstracts based on predefined inclusion and exclusion criteria. They resolved conflicts by discussing the issues and reaching consensus or by involving a third, senior reviewer. In this stage, reviewers dually and independently screened 300 records that we randomly selected from our literature searches. The dually agreed upon inclusions and exclusions served as the training set for DistillerAI.

During stage 2, DistillerAI replaced one human screener and provided prediction scores about inclusions or exclusions for all remaining records. The second human reviewer was not aware of predictions and screened the remaining abstracts. In stage 3, a second human reviewer resolved conflicts in decisions between the human screener and DistillerAI.

### Training DistillerAI

For each of the five screening teams, we randomly selected 300 abstracts as training sets from the database of our literature searches. Decisions about inclusions or exclusions of records in the training sets served as information for DistillerAI to build an algorithm for predictions. The manual for DistillerAI recommends 300 records as the optimal size for training sets based on internal simulation studies.

To reduce the risk of not having any included RCTs in the training set by chance, we employed weighted sampling to ensure that each training set included at least 5 of the 42 relevant studies of the AHRQ report.

After screeners had completed the training sets, we employed DistillerAI’s test function. The test function randomly selects records from the training set to determine the accuracy of predictions by comparing prediction scores to decisions about inclusion or exclusion in the training set. For each training set, we used the test function 5 times at a ratio of 80 to 20 (i.e., DistillerAI learns from 80% of the training set and predicts the randomly selected 20%). For all five training sets, DistillerAI’s naïve Bayesian approach provided better predictions than the support vector machine classifier. The mean accuracy score across the five training sets for the naive Bayesian approach was 87.9% compared with 47.6% for the support vector machine classifier. Consequently, we used DistillerAI’s naïve Bayesian approach for predictions for all five screening teams. Abstracts with prediction scores of 0.5 or greater were included; abstracts with prediction scores below 0.5 were excluded. A prediction score of 0.5 reflects a neutral prediction (i.e., DistillerAI cannot decide whether inclusion or exclusion is more likely). We chose a prediction score of 0.5 as a conservative threshold that would guarantee high sensitivity.

### Outcomes

We assessed three outcomes:

- Proportion of included abstracts. This outcome provides information about the number of full texts that need to be retrieved and reviewed, which has a substantial impact on the subsequent workload during the full-text review stage. We used the number of unscreened records (*n* = 2172) after completion of the training set as a denominator for all calculations; in other words, we did not include results of the training sets in any of the metrics.
- Proportion of conflicts and interrater agreement between human reviewers and DistillerAI. This outcome summarizes the agreement and the number of conflicts between human reviewers and DistillerAI, which had to be resolved by a second human reviewer. The number of unscreened records (*n* = 2172) served as the denominator for all calculations. We also determined the interrater agreement (prevalence-adjusted bias-adjusted kappa) between human screeners and DistillerAI.
- Accuracy of correctly classifying relevant and irrelevant studies. We determined sensitivities in identifying the 42 included studies of the reference standard as relevant. We also calculated specificities and areas under the receiver operating characteristics (ROC) curve.

### Comparisons and quantitative analyses

We assessed the above-mentioned outcomes for three abstract screening approaches:

1. The machine-assisted screening approach (as outlined in Fig. 1),
2. Single-reviewer screening (i.e., no DistillerAI involvement), and
3. Screening with DistillerAI alone (i.e., no human screener involvement after training DistillerAI).

For measures of accuracy, we organized results in 2 × 2 tables to determine true-positive, false-positive, true-negative, and false-negative decisions. We calculated sensitivities, specificities, and areas under the ROC curve with their 95% confidence intervals. For DistillerAI, we also calculated the ROC curve in an exploratory analysis using different prediction scores as thresholds. We conducted all quantitative analyses with Stata 13.1 (Stata Corporation, College Station, TX, USA).

## Results

Literature searches rendered 2472 references after deduplication. The 42 relevant randomized controlled trials (RCTs) of the reference standard compared second-generation antidepressants with nonpharmacological treatment options during acute-phase treatment of a major depressive disorder. Nonpharmacological interventions included various psychotherapies, acupuncture, St. John’s wort, omega-3-fatty acid, physical exercise, and S-adenosyl-L-methionine. Many of the available trials had serious methodological limitations. Authors of the reference report rated 16 of the 42 trials as high risk of bias and only 4 as low risk of bias [19].

As described in the “Methods” section, each of the five teams dually screened 300 randomly selected records to provide training sets for DistillerAI. The number of included studies (true positives) sampled into the training sets ranged from 10 to 16. In the following sections, we present results of the machine-assisted screening approach (as outlined in Fig. 1) and contrast them with single-reviewer screening (i.e., no DistillerAI involvement) or screening with DistillerAI only (no human screener involvement after training DistillerAI).

Table 2 provides a summary of various performance measures. Denominators for calculations of performance measures in the table vary by screening team because they discount for relevant studies that had been sampled into the training sets.

**Table 2:** Different performance measures for the machine-assisted screening approach, single-reviewer screening, and screening with DistillerAI alone

	Sensitivity (95% CI)	Specificity (95% CI)	Area under the curve (95% CI)	*N* of missed studies (proportion)	*N* of included abstracts (proportion)	*N* of conflicts (proportion)	*N* of included studies in training set
Team 1							
Machine-assisted screening	0.78 (0.59 to 0.90)	0.96 (0.96 to 0.97)	0.87 (0.80 to 0.95)	7/32 (22%)	97/2172 (4%)	126/2172 (6%)	10/300
Single-reviewer screening	0.78 (0.59 to 0.90)	0.96 (0.95 to 0.97)	0.87 (0.80 to 0.94)	7/32 (22%)	110/2172 (5%)		
DistillerAI screening	0.03 (0.00 to 0.21)	0.99 (0.98 to 0.99)	0.51 (0.48 to 0.54)	31/32 (97%)	27/2172 (1%)		
Team 2							
Machine-assisted screening	0.89 (0.70 to 0.97)	0.92 (0.91 to 0.93)	0.90 (0.84 to 0.96)	3/27 (11%)	232 /2172 (11%)	226/2172 (10%)	15/300
Single-reviewer screening	0.89 (0.69 to 0.97)	0.91 (0.89 to 0.92)	0.90 (0.84 to 0.96)	3/27 (11%)	221/2172 (10%)		
DistillerAI screening	0.00	0.99 (0.99 to 0.99)	0.50 (0.49 to 0.50)	27/27 (100%)	18/2172 (1%)		
Team 3							
Machine-assisted screening	0.65 (0.44 to 0.82)	0.96 (0.95 to 0.97)	0.81 (0.71 to 0.90)	9/26 (35%)	130/2172 (6%)	100/2172 (5%)	16/300
Single-reviewer screening	0.65 (0.44 to 0.82)	0.96 (0.95 to 0.97)	0.81 (0.71 to 0.90)	9/26 (35%)	104/2172 (5%)		
DistillerAI screening	0.23 (0.10 to 0.44)	0.99 (0.98 to 0.99)	0.61 (0.53 to 0.69)	20/26 (77%)	30/2172 (1%)		
Team 4							
Machine-assisted screening	0.86 (0.66 to 0.95)	0.94 (0.93 to 0.95)	0.90 (0.83 to 0.96)	4/28 (14%)	199/2172 (9%)	194/2172 (9%)	14/300
Single-reviewer screening	0.82 (0.62 to 0.93)	0.93 (0.92 to 0.94)	0.88 (0.80 to 0.95)	5/28 (18%)	165/2172 (8%)		
DistillerAI screening	0.32 (0.17 to 0.52)	0.97 (0.96 to 0.98)	0.65 (0.56 to 0.73)	19/28 (68%)	69/2172 (3%)		
Team 5							
Machine-assisted screening	0.74 (0.55 to 0.87)	0.95 (0.94 to 0.96)	0.84 (0.77 to 0.92)	8/31 (26%)	187/2172 (9%)	181/2172 (8%)	11/300
Single-reviewer screening	0.74 (0.55 to 0.87)	0.95 (0.94 to 0.95)	0.84 (0.77 to 0.92)	8/31 (26%)	138/2172 (6%)		
DistillerAI screening	0.13 (0.05 to 0.31)	0.97 (0.96 to 0.98)	0.55 (0.49 to 0.61)	27/31 (87%)	65/2172 (3%)		
Combined							
Machine-assisted screening	0.78 (0.66 to 0.90)	0.95 (0.92 to 0.97)	0.87 (0.83 to 0.90)	6/30 (22%)	8%	165/2172 (8%)	13/300
Single-reviewer screening	0.78 (0.66 to 0.89)	0.94 (0.91 to 0.97)	0.86 (0.82 to 0.89)	6/30 (22%)	7%		
DistillerAI screening	0.14 (0.00 to 0.31)	0.98 (0.97 to 1.00)	0.56 (0.53 to 0.59)	25/30 (86%)	2%		

*CI* = confidence interval; *N* = number

### Proportion of included abstracts

On average, the five screening teams using the machine-assisted approach included 8% (*n* = 174) of screened abstracts (range 4 to 11% [*n* = 87 to 239]). Single-reviewer screening, on average, included a similar proportion of abstracts as the machine-assisted approach (7% [*n* = 152]; range 5 to 10% [*n* = 109 to 217]). By comparison, DistillerAI, on average, rated only 2% (*n* = 43; range 1 to 3% [*n* = 22 to 65]) of screened abstracts as relevant for inclusion. The reference standard systematic review included 10% of screened abstracts.

### Proportion of conflicts and interrater agreement between human screeners and DistillerAI

Across the five screening teams, decisions about inclusion or exclusion resulted in conflicts between the human screeners and DistillerAI in 8% (*n* = 174; range 5 to 10% [*n* = 109 to 217]) of screened abstracts. In the majority of cases, the second human reviewers who resolved these conflicts confirmed the decisions of the human screeners. The interrater agreement between human screeners and DistillerAI with a prevalence-adjusted kappa was 0.85 (95% confidence interval [CI], 0.84 to 0.86).

### Accuracy of correctly classifying relevant and irrelevant studies

**Fig. 2:**
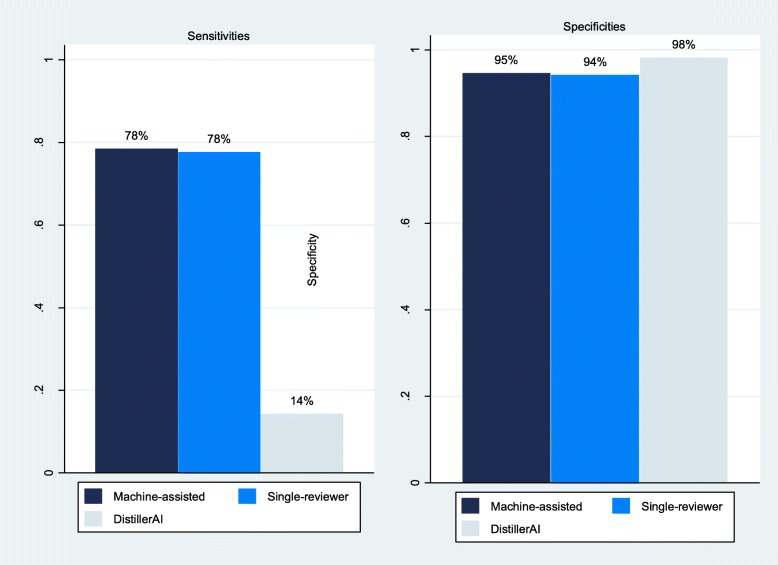
Sensitivities and specificities of machine-assisted screening, single-reviewer screening, and screening with DistillerAI alone

The most important outcome for the assessment of the performance of the machine-assisted screening approach is the sensitivity to identify correctly the 42 included studies of the reference standard review. The combined sensitivity of the machine-assisted screening approach was 78% (95% CI, 66 to 90%). In other words, the machine-assisted screening approach missed, on average, 22% of relevant studies. Of the 42 included studies of the reference standard review, the machine-assisted screening teams collectively missed 23 studies at least once (false-negative decisions; see Additional file 1). Figure 2 contrasts the sensitivity and specificity of the machine-assisted screening approach with the sensitivities and specificities of single-reviewer screening and screening with DistillerAI without human involvement. Overall, sensitivities of the machine-assisted approach and single-reviewer screening were substantially higher than the sensitivity of DistillerAI (78% vs. 78% vs. 14%; Fig. 2 and Table 2). On average, the machine-assisted screening approach and single-reviewer screening missed 22% of relevant studies compared with 86% of relevant studies that DistillerAI missed.

The specificity of the machine-assisted screening approach was 95% (95% CI, 92 to 97%). Specificities were similar between the machine-assisted approach, single-reviewer screening, and DistillerAI (95% vs. 94% vs. 98%; Fig. 2).

Table 2 also presents the areas under the curve, which summarizes the discriminative abilities of the approaches to distinguish relevant from irrelevant records. Machine-assisted screening and single-reviewer screening had similar areas under the curve (0.87 and 0.86, respectively); by contrast, DistillerAI was just slightly better than chance (0.56).

### Performance of DistillerAI for different prediction thresholds

**Fig. 3:**
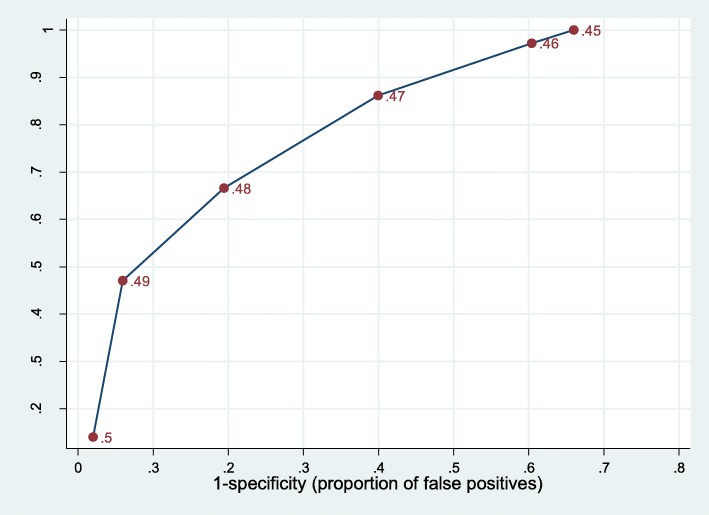
Receiver operating characteristics curve for DistillerAI

Because of the poor performance of DistillerAI with a threshold of 0.5, we further explored the accuracy of DistillerAI for thresholds below 0.5. Prediction scores below 0.5 indicate a greater probability that a record is irrelevant than relevant. Figure 3 presents the receiver operating characteristics (ROC) curve for DistillerAI for prediction scores between 0.5 and 0.45. To achieve a sensitivity close to 100%, the specificity would have to be reduced to 35% using a prediction score of 0.45. In other words, based on our sample, DistillerAI would have to include 65% of all abstracts to detect all relevant studies that were included in the reference standard review.

## Discussion

The objective of our methods study was to assess the accuracy of a machine-assisted abstract screening approach that temporarily replaces a human reviewer with a semi-automated screening tool (DistillerAI). The results of our project rendered a mean sensitivity of 78% and a mean specificity of 95% for this approach. The area under the ROC curve was 0.87.

Although the area under the ROC curve indicates adequate discriminative ability, the performance of the machine-assisted abstract screening approach is less than optimal for use in systematic reviews. During an abstract screening in systematic reviews, false-negative decisions (i.e., excluding relevant records) are more consequential than false-positive decisions (i.e., including irrelevant records). The subsequent full-text review will rectify false-positive decisions without consequences for the validity of a systematic review. By contrast, false-negative decisions might cause relevant records to be omitted, which could affect the validity of a systematic review. A machine-assisted screening approach that misses 22% of relevant studies, therefore, is not adequate for systematic reviews.

Several factors might have contributed to the poor sensitivity of the machine-assisted screening approach in our study. First, the choice of the topic probably had a substantial impact on the performance of the approach. The comparative effectiveness of pharmacological and nonpharmacological treatments comprises a wide spectrum of interventions, particularly of nonpharmacological interventions. The Cochrane Common Mental Disorders group, for example, lists more than 80 psychological interventions for the treatment of depression. A less complex topic might have led to a better performance of DistillerAI and different conclusions. Systematic reviews, however, are often multi-faceted and complex. Using an unrealistically simple topic or an artificially clean dataset might have overestimated the performance under real-world conditions. Second, many of the published studies, particularly on complementary and alternative treatments, were conducted in countries where English is not the native language. Some of these abstracts were difficult to understand and interpret, which was also a contributing factor to the screening teams dually and falsely excluding five relevant studies during the screening of training sets. A partially incorrect training set is not an optimal precondition for testing the performance of machine-assisted abstract screening but might reflect real-life conditions. Nevertheless, incorrect decisions of human screeners had no apparent impact on the sensitivity of DistillerAI. For example, the team with the highest sensitivity of DistillerAI (team 4: 0.32) falsely excluded two out of 14 relevant studies in the training set. In screening teams without false-negative decisions in the training set, sensitivities ranged from 0.03 to 0.23 (see Table 2). Third, we adhered to DistillerAI’s recommendation regarding the optimal sample size for training sets (*n* = 300). This recommendation is based on simulation studies and might have been too small to adequately train DistillerAI for our topic. The small training sets might also explain why the naïve Bayesian approach consistently provided better results than the support vector machine classifier.

Taken together, these issues might have contributed to a machine-learning phenomenon called “hasty generalizations.” This term describes situations in which the training set is not fully representative of the remaining records [5]. Given the broad and complex topic, hasty generalizations might have played a role despite the attempt to ensure the generalizability of the training sets with random sampling.

The performance of DistillerAI, in general, was disappointing. The average sensitivity was 0.14; in one case DistillerAI missed all relevant studies. Adding DistillerAI to single-reviewer screening did not provide additional gains in accuracy but instead created conflicts between human screeners and DistillerAI in 5 to 10% of records. These conflicts had to be resolved by a second human screener, which required effort without a gain in accuracy. In other words, DistillerAI did not improve the proportion of incorrect decisions that human screeners made when they screened abstracts. The ROC curve of DistillerAI implies that lowering the prediction threshold to 0.45 would have achieved a sensitivity close to 100%. With a prediction score of 0.45, however, the specificity would have decreased to 35%, which in turn would have caused a substantial increase in the number of conflicts between human screeners and Distiller AI because DistillerAI would have included about 65% of abstracts.

Our study has several strengths and weaknesses. A strength is that we used five teams who screened the same abstracts in parallel. Using five screening teams mitigated errors and subjective decisions of individual screeners, as well as the influence of screening experience and content expertise on results. Another strength of our study is that we mimicked a real-world abstract screening situation, including unintended incorrect decisions that human screeners made when they reviewed the training sets. To minimize selection bias, we randomly selected records for the training sets. Such an approach reflects real-world conditions under which machine-assisted screening would take place. We purposely did not use decisions from the reference standard dataset to train DistillerAI. The final included and excluded studies of a systematic review are the results of a process that leverages more than the decisions of two screeners. The final body of evidence is also a result of feedback from the review team, review of reference lists of other systematic reviews, and comments from external peer reviewers. Finally, the choice of our reference standard is also a strength of our study. Our reference standards were the final included and excluded studies and not decisions during the title and abstract screening of the reference review. Decisions during abstract screening are an insufficient reference standard because screening decisions among screening teams can vary substantially. It is conceivable that a semi-automated screening tool makes more precise screening decisions than human screeners make but would end up with inferior accuracy because of the imperfect reference standard.

A weakness of our study is that we employed a focused, stepwise literature search to recreate the evidence base for one Key Question of a systematic review. In other words, we knew from the outset which studies were relevant for the topic and tailored the searches accordingly. Our searches, therefore, presumably produced less noise than a regular systematic literature search. The spectrum and the ratio of relevant and irrelevant records were most likely different than those in a de novo regular systematic literature search. An additional weakness of our study is the complex topic. The results of our study are also not generalizable to other semi-automated literature screening tools. Furthermore, when we calculated accuracy measures, we assumed that falsely excluded studies would be missed by the review. In reality, a systematic review has subsequent processes in place that can detect incorrectly excluded records later during the review process, such as review of reference lists of other systematic reviews or external peer review. It is conceivable that some of the studies missed during abstract screening would ultimately still be included in the final systematic review. Finally, although our outcome measures provide a comprehensive picture of the accuracy and the performance of the machine-assisted screening approach, they also have limitations. We do not know whether falsely excluded studies would change the conclusions of the systematic review. This is particularly relevant for users of rapid reviews who are willing to accept that the review misses relevant studies. For them, it is more important whether conclusions would change because of missed studies. A recent international survey showed that decision-makers are willing to accept up to 10% of incorrect wrong conclusions in exchange for a rapid evidence product [20].

In a recent commentary, O’Connor and colleagues explored reasons for the slow adoption of automation tools [21]. They argue that the adoption of such tools requires credible evidence that automation tools are non-inferior or even superior in accuracy compared with standard practice. Our study provides evidence that non-inferiority is clearly not the case yet for DistillerAI. Few other studies have assessed semi-automated screening tools under real-world conditions [5, 15, 16]. The results of these studies are consistent with our findings that semi-automated screening tools have the potential for expediting reviews but that the accuracy is still limited [15, 16].

Future studies need to explore whether semi-automated screening tools could prove useful in identifying records that are clearly not relevant, which is a different approach than we took in our study. Future studies also need to assess the comparative accuracy of different screening tools under pragmatic, real-world screening situations. A still unanswered question is also how semi-automated screening tools perform when used with abbreviated literature searches that have a higher specificity than comprehensive systematic literature searches. Waffenschmidt et al., for example, proposed an abbreviated search strategy for RCT randomized controlled trials [22]. This approach combines a simple-structured Boolean search in PubMed with searches using the “similar articles” function in PubMed. In a case study, this approach reduced the number of abstracts that needed to be screened by up to 90% without missing studies that would have changed conclusions [23]. It is conceivable that such a targeted literature search approach could improve the performance of semi-automated screening tools because they would have to deal with less noise.

## Conclusions

Systematic reviews require substantial human effort for often repetitive and labor-intensive tasks. Automation to assist reviewers during systematic reviews becomes increasingly viable. The findings of our study imply that the accuracy of DistillerAI is not yet adequate to replace a human screener temporarily during abstract screening. The approach that we tested missed too many relevant studies and created too many conflicts between human screeners and DistillerAI. Rapid reviews, which do not require detecting the totality of the relevant evidence, may find semi-automation tools to have greater utility than traditional reviews.

## Supplementary information

**Additional file 1.** Characteristics of studies that machine-assisted screening teams missed at least once.

## Data Availability

The datasets analyzed during the current study are available from the corresponding author on reasonable request.

## Abbreviations

AHRQ: Agency for Healthcare Research and Quality
CI: Confidence interval
RCT: Randomized controlled trial
ROC: Receiver operating characteristics
