# Supplementary material for: Assessing the accuracy of machine-assisted abstract screening with DistillerAI: a user study
Source: Syst Rev. 2019 Nov 15;8:277. doi: 10.1186/s13643-019-1221-3 (PMC6857277; doi:10.1186/s13643-019-1221-3)
Supplement: Supplementary file 1 — Additional file 1. Characteristics of studies that machine-assisted screening teams missed at least once. [file 13643_2019_1221_MOESM1_ESM.docx]

Supplementary Files

**Supplementary File 1. Characteristics of studies that machine-assisted screening teams missed at least once**

| **Author and Year** | **Intervention** | **Sample Size, Risk of Bias** | **Falsely Excluded by:** | | | | |
| --- | --- | --- | --- | --- | --- | --- | --- |
|  |  |  | Team 1 | Team 2 | Team 3 | Team 4 | Team 5 |
| Barber et al., 2012(1) | Psychotherapy | N=106, Medium |  |  |  | X |  |
| Bastos et al., 2013(2) | Psychotherapy | N=272, Medium |  |  |  | X | X |
| Blom 2007(3) | Psychotherapy | N=207, Medium |  | X |  |  |  |
| Blumenthal et al., 2007(4) | Exercise | N=153, Medium |  |  | X |  |  |
| Frank et al., 2011(5) | Psychotherapy | N=318, High | X |  | X |  |  |
| Gastpar et al., 2005(6) | St. John’s wort | N=241, Medium |  | X |  |  |  |
| Gertsik et al., 2012(7) | Omega-3 fatty acid augmentation of citalopram treatment | N=42, High |  |  |  | X |  |
| Hegerl et al., 2010(8) | Psychotherapy | N=48, Medium |  |  |  |  | X |
| Huang et al., 2005(9) | Electro-scalp acupuncture | N=98, Medium | X | X | X |  |  |
| Jazayeri et al., 2008(10) | Omega-3 fatty acid eicosapentaenoic acid | N=48, High |  |  |  |  | X |
| Kennedy et al., 2007(11) | Psychotherapy | N=31, High | X |  |  |  | X |
| Lam et al., 2013(12) | Psychotherapy | N=80, Medium |  | X | X |  | X |
| McGrath et al., 2013(13) | Psychotherapy | N=82, High | X |  |  | X | X |
| Menchetti et al., 2014(14) | Psychotherapy | N=287, Medium |  |  | X |  | X |
| Mischoulon et al., 2014(15) | Eicosapentaenoic acid | N =189, High |  |  | X |  |  |
| Mynors-Wallis et al., 2000(16) | Psychotherapy | N=151, Medium |  |  | X |  |  |
| Raue et al., 2009(17) | Psychotherapy | N=60, High | X | X |  |  | X |
| Schrader et al., 2000(18) | St. John’s wort | N=106, Medium |  |  |  |  | X |
| Segal et al., 2006(19) | Psychotherapy | N=301, High | X |  |  |  |  |
| Song et al., 2007(20) | Electroacupuncture | N=90,  High | X |  | X |  |  |
| Zhang et al., 2009(21) | Acupuncture | N=80, Medium |  |  | X |  |  |

N = number of study participants.

1. Barber JP, Barrett MS, Gallop R, Rynn MA, Rickels K. Short-term dynamic psychotherapy versus pharmacotherapy for major depressive disorder: a randomized, placebo-controlled trial. J Clin Psychiatry. 2012;73(1):66-73.

2. Bastos AG, Guimaraes LS, Trentini CM. Neurocognitive changes in depressed patients in psychodynamic psychotherapy, therapy with fluoxetine and combination therapy. J Affect Disord. 2013;151(3):1066-75.

3. Blom MB, Jonker K, Dusseldorp E, Spinhoven P, Hoencamp E, Haffmans J, et al. Combination treatment for acute depression is superior only when psychotherapy is added to medication. Psychother Psychosom. 2007;76(5):289-97.

4. Blumenthal JA, Babyak MA, Doraiswamy PM, Watkins L, Hoffman BM, Barbour KA, et al. Exercise and pharmacotherapy in the treatment of major depressive disorder. Psychosom Med. 2007;69(7):587-96.

5. Frank E, Cassano GB, Rucci P, Thompson WK, Kraemer HC, Fagiolini A, et al. Predictors and moderators of time to remission of major depression with interpersonal psychotherapy and SSRI pharmacotherapy. Psychol Med. 2011;41(1):151-62.

6. Gastpar M, Singer A, Zeller K. Efficacy and tolerability of hypericum extract STW3 in long-term treatment with a once-daily dosage in comparison with sertraline. Pharmacopsychiatry. 2005;38(2):78-86.

7. Gertsik L, Poland RE, Bresee C, Rapaport MH. Omega-3 fatty acid augmentation of citalopram treatment for patients with major depressive disorder. J Clin Psychopharmacol. 2012;32(1):61-4.

8. Hegerl U, Hautzinger M, Mergl R, Kohnen R, Schütze M, Scheunemann W, et al. Effects of pharmacotherapy and psychotherapy in depressed primary-care patients: a randomized, controlled trial including a patients' choice arm. Int J Neuropsychopharmacol. 2010;13(1):31-44.

9. Huang Y, Htut W, Li D, Tang A, Li Q, Shi N, et al. Studies on the clinical observation and cerebral glucose metabolism in depression treated by electro-scalp acupuncture compared to fluoxetine. International Journal of Clinical Acupuncture. 2005;14(1):7-26.

10. Jazayeri S, Tehrani-Doost M, Keshavarz SA, Hosseini M, Djazayery A, Amini H, et al. Comparison of therapeutic effects of omega-3 fatty acid eicosapentaenoic acid and fluoxetine, separately and in combination, in major depressive disorder. Aust N Z J Psychiatry. 2008;42(3):192-8.

11. Kennedy SH, Konarski JZ, Segal ZV, Lau MA, Bieling PJ, McIntyre RS, et al. Differences in brain glucose metabolism between responders to CBT and venlafaxine in a 16-week randomized controlled trial. Am J Psychiatry. 2007;164(5):778-88.

12. Lam RW, Parikh SV, Ramasubbu R, Michalak EE, Tam EM, Axler A, et al. Effects of combined pharmacotherapy and psychotherapy for improving work functioning in major depressive disorder. Br J Psychiatry. 2013;203(5):358-65.

13. McGrath CL, Kelley ME, Holtzheimer PE, Dunlop BW, Craighead WE, Franco AR, et al. Toward a neuroimaging treatment selection biomarker for major depressive disorder. JAMA psychiatry. 2013;70(8):821-9.

14. Menchetti M, Rucci P, Bortolotti B, Bombi A, Scocco P, Kraemer HC, et al. Moderators of remission with interpersonal counselling or drug treatment in primary care patients with depression: randomised controlled trial. Br J Psychiatry. 2014;204(2):144-50.

15. Mischoulon D, Price LH, Carpenter LL, Tyrka AR, Papakostas GI, Baer L, et al. A double-blind, randomized, placebo-controlled clinical trial of S-adenosyl-L-methionine (SAMe) versus escitalopram in major depressive disorder. J Clin Psychiatry. 2014. Apr;75(4):370-6

16. Mynors-Wallis LM, Gath DH, Day A, Baker F. Randomised controlled trial of problem solving treatment, antidepressant medication, and combined treatment for major depression in primary care. BMJ. 2000;320(7226):26-30.

17. Raue PJ, Schulberg HC, Heo M, Klimstra S, Bruce ML. Patients' depression treatment preferences and initiation, adherence, and outcome: a randomized primary care study. Psychiatr Serv. 2009;60(3):337-43.

18. Schrader E. Equivalence of St John's wort extract (Ze 117) and fluoxetine: a randomized, controlled study in mild-moderate depression. Int Clin Psychopharmacol. 2000;15(2):61-8.

19. Segal ZV, Kennedy S, Gemar M, Hood K, Pedersen R, Buis T. Cognitive reactivity to sad mood provocation and the prediction of depressive relapse. Arch Gen Psychiatry. 2006;63(7):749-55.

20. Song Y, Zhou D, Fan J, Luo H, Halbreich U. Effects of electroacupuncture and fluoxetine on the density of GTP-binding-proteins in platelet membrane in patients with major depressive disorder. J Affect Disord. 2007;98(3):253-7.

21. Zhang WJ, Yang XB, Zhong BL. Combination of acupuncture and fluoxetine for depression: a randomized, double-blind, sham-controlled trial. J Altern Complement Med. 2009;15(8):837-44.
